# Therapeutic hyperthermia promotes lipid export and HSP70/90 during machine perfusion of human livers

**DOI:** 10.14814/phy2.70348

**Published:** 2025-05-09

**Authors:** Adam M. Thorne, Yana Geng, Veerle A. Lantinga, Marieke Smit, Jan Albert Kuivenhoven, Robert J. Porte, Folkert Kuipers, Peter Olinga, Justina C. Wolters, Vincent E. de Meijer

**Affiliations:** ^1^ Department of Liver Transplantation and HPB Surgery University of Groningen and University Medical Center Groningen Groningen The Netherlands; ^2^ Department of Pharmaceutical Technology and Biopharmacy University of Groningen Groningen The Netherlands; ^3^ Department of Pediatrics, University of Groningen University of Groningen and University Medical Center Groningen Groningen The Netherlands; ^4^ Erasmus MC Transplant Institute, Department of Surgery, Division of HPB and Transplant Surgery University Medical Center Rotterdam Rotterdam The Netherlands; ^5^ European Research Institute for the Biology of Ageing (ERIBA) University of Groningen and University Medical Center Groningen Groningen The Netherlands

**Keywords:** hyperthermia, lipids, liver, machine perfusion, proteomics

## Abstract

Liver transplantation is the only curative option for end‐stage liver disease. Donor shortages necessitate the use of higher risk donor livers, including fatty livers, which are more susceptible to ischemia‐reperfusion injury. Machine perfusion has improved graft utilization and is typically performed at hypothermic (8–12°C) or normothermic (35–37°C) temperatures. Here we studied the impact of mild hyperthermia (40°C) as a therapeutic intervention for fatty livers using in‐depth proteomic and lipoprotein profiling of whole organ perfusion and precision‐cut liver slices. We observed proteomic changes with metabolic alterations over time, evidenced by a significant increase in lipid export in whole organ perfusions. Furthermore, PCLS showed significant upregulation of metabolic processes and heat shock protein response after 24 h of hyperthermia. Machine perfusion under hyperthermic conditions may be a potential strategy to improve the utilization of fatty liver grafts, ultimately expanding the donor pool and improving transplant outcomes.

## Graphical abstract

Proteomic and lipoprotein analysis demonstrates that mild hyperthermia (40°C) during machine perfusion enhances lipid export and upregulates heat shock protein responses in human donor livers.

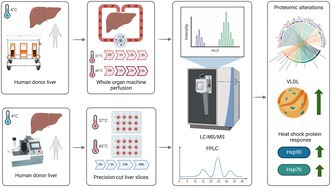

## 1 INTRODUCTION

Liver transplantation remains the only curative option for patients with end‐stage liver disease today. However, the demand for suitable donor livers far exceeds the current supply, creating a persistent global organ shortage, with waiting list mortality approaching 20% (Eurotransplant, n.d.). This necessitates the use of high‐risk, so‐called ‘extended‐criteria donor’ (ECD) livers, including donation after circulatory death (DCD) donors and grafts with varying degrees of steatosis, for transplantation (Orman et al., 2015). Effective preservation of these ECD grafts is essential to ensure safe and complication‐free outcomes post‐transplantation.

Metabolic‐associated steatotic liver disease (MASLD), ranging from simple steatosis to end‐stage cirrhosis, is a hallmark indication of metabolic syndrome and the most common chronic liver disease, with an estimated global prevalence of 25% (Asrani et al., 2019; Diehl & Day, 2017). Furthermore, fatty liver‐induced cirrhosis and associated hepatocellular carcinoma are becoming leading indications for liver transplantation worldwide (Asrani et al., 2019; Diehl & Day, 2017; Shingina et al., 2019). As demand for liver transplantation increases, so does the number of donor livers with steatosis due to the ongoing obesity epidemic. ECD organs, particularly those with high fat content, have impaired tolerance to ischemia–reperfusion injury (IRI) due to abnormal microcirculation secondary to compression of sinusoids by intracellular lipid droplets, decreased adenosine triphosphate (ATP) stores and ATP regeneration, and increased susceptibility to oxidative stress (Abu‐Amara et al., 2010). MASLD can be reversed by weight loss and dietary changes (Bhat et al., 2012; Sacks et al., 2009). Aggressive lifestyle changes in living liver donors have resulted in substantial defatting of the liver (Choudhary et al., 2015). However, in liver transplantation, the time window to recondition fatty livers from deceased donors is limited to hours, whereas dietary and pharmacologic treatments take weeks or months.

Traditionally, static cold storage (SCS) has been the preservation method of choice for organ transplantation. Cold preservation reduces metabolic demand in the organ, helping to prevent ischemic cellular damage. However, SCS remains an insufficient preservation method for ECD liver grafts, and alternative interventions to recondition fatty livers shortly before transplantation are urgently required (Boteon et al., 2018).

To address this, ex‐situ machine perfusion has been developed as a technique to effectively preserve and functionally assess donor livers prior to transplant. Typically, ex‐situ machine perfusion occurs at two temperatures: (dual) hypothermic oxygenated machine perfusion ([D]HOPE; 8–12°C) and normothermic machine perfusion (NMP; 37°C) (de Meijer et al., 2019). Like SCS, DHOPE reduces liver metabolism, but it also resuscitates the perfused liver by supplying oxygen. This, in turn, restores cellular ATP to support aerobic metabolism upon reperfusion in the recipient and has been shown to significantly reduce post‐transplant complications (van Rijn et al., 2021). With NMP, the liver becomes metabolically active, providing a platform for assessment of hepatocellular and cholangiocellular processes, thus allowing functional evaluation of the graft (Matton et al., 2019; Nasralla et al., 2018; van Leeuwen et al., 2022). Despite these advancements, steatotic livers remain risky due to persistent pre‐, peri‐, and post‐transplant complications (Patrono et al., 2023). Active metabolism is essential for liver defatting, but studies on the effect of machine perfusion to recondition human fatty liver grafts are limited (Da Silva et al., 2023). One approach to further enhance the metabolic rate of the liver is to increase the temperature to mild hyperthermic conditions (40–41.5°C). This increase could lead to improved lipid export, better vascular compliance, and induction of heat shock proteins, which may mitigate the harmful effects of IRI (Thorne et al., 2020). While there has been extensive research into machine perfusion techniques from cold to normothermic temperatures, the application of hyperthermic conditions has primarily been limited to clinical interventions in cancer treatments in both humans and animals (Chang et al., 2018; Kimball et al., 2018; Laupland, 2009). Machine perfusion of the liver at hyperthermic conditions (HyMP) remains understudied.

From this, perfusion of human donor livers at mild hyperthermic temperatures (40°C) emerges as a potential preservation strategy for ECD grafts (Thorne et al., 2020). We hypothesized that machine perfusion at mild hyperthermic temperatures may increase the metabolic rate, leading to improved mobilization and utilization of intracellular fat, and mitigation of IRI by inducing protective mechanisms such as heat shock protein (HSP) responses (Archer et al., 2018; Mokuno et al., 2004). Through systematic evaluation of these factors in human whole organ machine perfusion and precision‐cut liver slice (PCLS) models, here we study the benefits of hyperthermic perfusion and its potential to enhance the quality of donor livers, thereby expanding the donor pool and improving transplant outcomes.

## 2 MATERIALS AND METHODS

### 2.1 Whole organ procurement and machine perfusion

**FIGURE 1:**
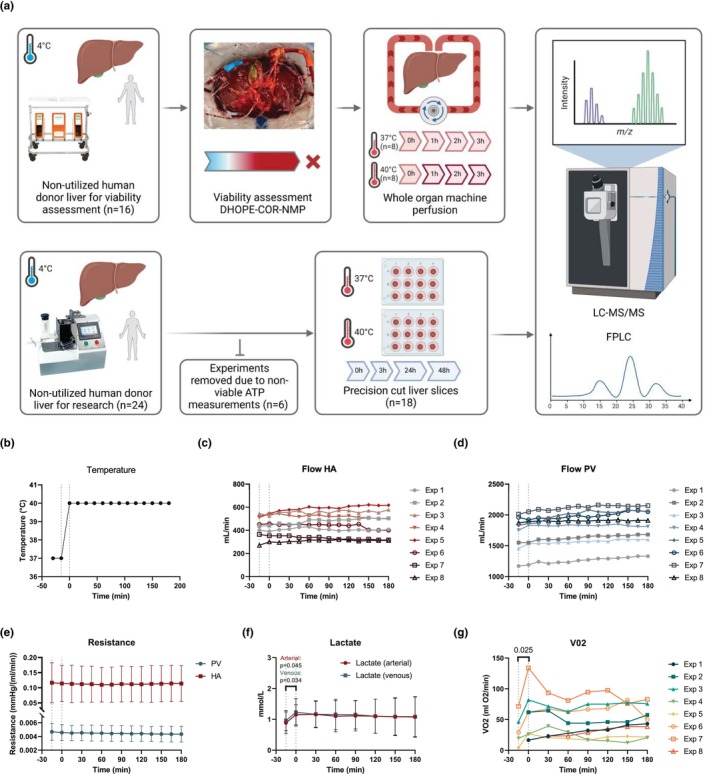
Overview schematic of mild hyperthermia in whole organ perfusion and precision‐cut liver slices (PCLS) models. (a) Whole organ perfusion (*n* = 16) was performed at 40°C (*n* = 8) for 3 h on clinical human donor livers following rejection for transplantation after viability assessment using DHOPE‐COR‐NMP. Control perfusions (*n* = 8) were performed at 37°C on livers accepted for transplantation following viability assessment. PCLS (*n* = 18) were cut from non‐utilized human donor organs available for research (with no prior machine perfusion) and incubated at 37°C or 40°C for up to 48 h. Proteins from tissue (whole organ and PCLS) and perfusate (whole organ) were then processed for LC–MS/MS analysis using data independent acquisition. Created with Biorender.com. (b) Temperature change protocol. (c) Flow for hepatic artery (HA). (d) Fow for portal vein (PV). (e) Hepatic resistance in HA and PV. (f) Lactate levels in arterial and venous samples. (g) Calculated oxygen consumption (VO_2_). Perfusion parameters of hepatic artery (HA) and portal vein (PV) for whole organ machine perfusion.

All donor livers included in this study were nationally declined for transplantation and were procured by one of the regional multi‐organ procurement teams in the Netherlands using a previously described standardized procedure (van Rijn et al., 2017). A mandatory 5‐minute no‐touch period after circulatory arrest was observed for all DCD donors. Following procurement, livers were transported to our center using SCS. Livers undergoing whole organ machine perfusion were initially accepted for viability assessment using NMP (van Leeuwen et al., 2022). If deemed not suitable for transplantation after testing, the liver was declined for transplantation but remained on the pump for application of mild hyperthermia (40°C) for an additional 3 h. Control samples (37°C) were obtained from livers that met all viability criteria and remained on the pump until transplantation. Livers being used for PCLS were specifically allocated to transplantation research, processed directly after transport to our center, and did not undergo a period of machine perfusion before incubation. An overview of the experimental workflow is depicted in Figure 1a.

Whole organ machine perfusion was carried out as previously described (van Leeuwen et al., 2022), using a Liver Assist device (XVIVO, Groningen, the Netherlands). As these livers had the potential to be transplanted following viability assessment, all livers underwent a standardized clinical machine perfusion protocol comprising at least 1 h of dual hypothermic oxygenated machine perfusion (DHOPE) using University of Wisconsin (UW) Machine Perfusion Solution (PumpProtect, Carnamedica, Warsaw, Poland) to mitigate reperfusion injury and reduce cold ischemic times (van Rijn et al., 2021). Following a perfusate switch to a red blood cell‐based solution, livers were rewarmed gradually from 20°C to 37°C over 1 h. Livers were then perfused for 2.5 h at 37°C (NMP), after which the viability assessment was made. Whole organ hyperthermia was performed on livers that did not meet the cholangiocellular viability criteria and were subsequently declined for transplantation due to presumed excessive injury to the biliary tree (van Leeuwen et al., 2022). All livers that subsequently underwent whole organ hyperthermia passed hepatocellular viability criteria, indicating sufficient parenchymal metabolic function. These livers remained on the perfusion device, where baseline parameters were taken at 37°C before increasing the temperature to 40°C for another 3 h (Figure 1b). Machine perfusion parameters such as portal vein and hepatic artery flows and pressures, as well as biochemical parameters including perfusate pco_2_, pH, and FiO_2_ were adjusted and maintained within normal NMP ranges as previously described (van Leeuwen et al., 2022). Tissue biopsies and perfusate samples were collected at baseline (0 h, 37°C) and every 1 h of hyperthermic or normothermic (control) perfusion. O_2_ consumption was calculated using the following equation:

$${\mathrm{VO}}_{2}\;\left(\mathrm{mL}\;O_{2}/\min\right)={\mathrm{DO}}_{2}–\left(Q_{\mathrm{VC}}\times C_{\mathrm{VC}}O_{2}\right)$$

With the absence of blood gas sampling from the PV side of the machine, assumptions were made for PV pco_2_ (HA pco_2_ + 1), po_2_ (6.5%), sO_2_ (85%) and HCO_3_ (HA HCO_3_ + 1). Where DO_2_ is the oxygen delivery of the hepatic artery and portal vein combined in O_2_/min, Q_VC_ is the blood flow from the vena cava in L/min, and C_VC_O_2_ is the oxygen content in mL O_2_/L in the vena cava.

### 2.2 Preparation of PCLS

Livers used for PCLS experiments were nationally declined and allocated for research without the chance for transplantation (e.g., because of a donor with active malignancy). Therefore, these livers did not undergo any type of machine perfusion prior to PCLS experiments. Upon arrival at our center (after SCS), core biopsies 6 mm in diameter were prepared from the liver with a biopsy punch. The core biopsies were then cut into slices approximately 250 μm thick (wet weight ~5 mg) using a Krumdieck tissue slicer (Alabama Research and Development, Munford, USA) according to a previously described protocol (de Graaf et al., 2010). PCLS were incubated in 12‐well plates (Greiner Bio‐One, Kremsmünster, Austria) with 3 mL culture media comprising Williams medium E supplemented with Glutamax (Invitrogen, Bleiswijk, the Netherlands), 50 μg/mL gentamycin (Invitrogen), and 25 mM glucose (Merck, Darmstadt, Germany) at 37°C or 40°C for 3, 24, or 48 h under 80% O_2_ and 5% CO_2_. The plates were incubated in a shaking incubator at 90 cycles/min. Media were changed every 24 h. PCLS tissue was collected at 0, 3, 24, and 48 h for analysis.

### 2.3 PCLS viability assessment

PCLS viability was determined at all time points using ATP content as previously described (de Graaf et al., 2010; Karsten et al., 2019). In brief, after incubation, PCLS were immediately snap frozen in 1 mL of sonication solution (70% ethanol and 2 mM ethylenediaminetetraacetic acid [EDTA], pH = 10.9) using liquid nitrogen and stored at −80°C until use. To extract ATP, PCLS were homogenized, the homogenate centrifuged (16,000×*g* at 4°C for 5 min), the supernatant removed, and the resulting pellet left to dry. An ATP bioluminescence assay kit (Roche Diagnostics, Mannheim, Germany) was used to determine ATP content in the supernatant. The leftover pellet was used to determine protein content using the Bio‐Rad DC Protein Assay (Bio‐Rad, Munich, Germany) for normalization of ATP levels between PCLS.

### 2.4 Histology

For morphological assessment at each timepoint, all whole organ biopsies and PCLS tissue were formalin‐fixed, paraffin‐embedded (FFPE) and cut into 4 μm sections. Sections were then stained with Hematoxylin and Eosin (H&E) stain.

### 2.5 Proteomics sample preparation

Tissue (whole organ and PCLS) and perfusate (whole organ) samples were prepared for mass spectrometry analysis using a single‐pot, solid‐phase‐enhanced sample‐preparation (SP3) protocol (Müller et al., 2020). Prior to protein digestion, perfusate samples underwent albumin depletion using isopropanol (IPA) with 1.0% trichloroacetic acid (TCA) (Liu et al., 2014). In brief, 20 μL of perfusate was mixed with 200 μL of IPA with 1% TCA solution and vortexed for 2 min. Samples were then centrifuged at 1500×*g* at 5°C for 5 min, and the supernatant discarded. The remaining pellets were washed with 200 μL methanol, air‐dried, and resuspended in 50 μL of 100 mM ammonium bicarbonate (ABC).

Tissue samples were lysed in RIPA buffer (Thermo Fischer Scientific, MA, USA) with added protease inhibitor cocktail (Roche, Switzerland), with volumes normalized to tissue weight (weight/volume ratio of 10 μg/300 μL). Tissue samples were mechanically lysed using a Precellys 24 (Bertin Technologies, France) with 2 × 20‐s bursts at 3600 rpm. Between each lysing cycle, samples were stored on ice for 5 min to avoid temperature increases. Protein concentrations of tissue lysate and albumin‐depleted perfusate were determined using Pierce BCA assay (Thermo Fisher Scientific, MA, USA). Sample volumes containing 10 μg protein were then diluted to 10 μg/25 μL using 100 mM ABC for tryptic digestion SP3 tryptic digestion using a Bravo automated liquid handling platform (Agilent, CA, USA). Equal volumes of Sera‐Mag Speed Beads A and B (hydrophilic/hydrophobic; Thermo Fisher Scientific, MA, USA) were washed twice in 4× and 5× volumes of 100 mM ABC and resuspended in 10× volume of 100 mM ABC to give a final concentration of 2 μg beads/10 μg protein (5:1 ratio). For washing and protein digestion, samples were first reduced using 20 mM dithiothreitol (DTT) at 60°C for 30 min and alkylated at room temperature in the dark for 30 min. Beads were then added to the samples and mixed for 5 min. Following this, wash steps of 2× 200 μL 80% ethanol and 180 μL acetonitrile were performed. After washing, solvents were removed and the beads resuspended in sequencing grade trypsin (Promega, WI, USA) at a trypsin‐to‐protein ratio of 1:100 and left to digest at 37°C overnight. The digestion was then quenched with 5 μL 1% formic acid (FA) and the beads were removed. Digested samples were then diluted with 100 mM ABC to a final concentration of 1 μg peptide/20 μL ABC for mass spectrometry analysis.

### 2.6 Mass spectrometry

Liquid chromatography (LC) tandem mass spectrometry (LC–MS/MS) was performed using an Orbitrap Exploris 480 quadrupole mass spectrometer connected to a non‐electrospray ion source (Thermo Fisher Scientific, MA, USA) coupled with a front‐end high field asymmetric waveform ion mobility (FAIMS) device (Thermo Fisher Scientific, MA, USA). Chromatographic separation of peptides was performed by LC on an EVOSEP One system using Pure tips (EVOSEP, Odense, Denmark; as per manufacturer's instructions) using an EVOSEP Performance 30 SPD column (ReproSil‐Pur C18, 1.5 μm beads, 15 cm × 150 μm) maintained at 40°C. In brief, 20 μL of sample with a peptide concentration of 1 μg/20 μL was loaded into the EVOSEP tips and peptides were separated using the 30 samples per day (SPD) manufacturer's program with a 44 min gradient. The mass spectrometer was operated in positive ion mode and data‐independent acquisition mode (DIA) using isolation windows of 12 m/z with a precursor mass range of 300–1200. The orbitrap resolution was set to 120,000 and DIA scan resolution at 30,000. Two compensation voltages (−60 V and −45 V) were used with the FAIMS device.

### 2.7 Lipid analyses

Enzymatic, colorimetric assays were used to measure total cholesterol, triglycerides (TGs) and non‐esterified fatty acids (FFA) in the whole organ perfusate. Assays were performed according to the manufacturers protocol for cholesterol (Diasys Diagnostic System GmbH, Cat. no. 113009910026) using cholesterol FS as a reference (Diasys Diagnostic System GmbH, Cat. no. 113003010030). Triglycerides (Diagnostic System GmbH; Cat. no. 157109910917) were measured using the standard Precimat Glycerol (Roche; Cat. no. 10166588) as a reference. For measuring FFA (Diasys Diagnostic System GmbH; Cat. no. 57819910935), Oleic acid was used as a reference (Diasys Diagnostic System GmbH; Cat. no. 157809910065). Apolipoprotein (apo)B was measured using an Atellica Nefelometer (Siemens Healthcare Diagnostics B.V.) at an absorbance of 840 nm. Pooled plasma samples were fractionated by fast‐performance liquid chromatography (FPLC), as previously described (Vos et al., 2023). In short, plasma lipoproteins are separated by size on a column, and an ultraviolet detector measures cholesterol concentrations via an enzymatic colorimetric reaction determination post‐column.

### 2.8 Statistical analysis

MS raw data were searched using Spectronaut software (version 18.6.231227, Biognosys) using library‐free DIA analysis workflows. Oxidation and deamidation were set as variable modifications, and carbamidomethylation was set as a fixed modification. Data were searched against human protein sequences using the UPR homoSapiens FASTA file (20,401 entries). MS1 values were used for protein quantitation and globally normalized. Quantitative protein values for identified proteins were analyzed using Perseus software (v1.6.15.0). Correction of potential batch effects was performed using the Limma algorithm. Volcano plots were generated in Perseus using log2 transformed data with imputation of missing values using normal distribution. Paired student's *t*‐tests with a permutation‐based FDR of 0.05 were used for multiple testing corrections, and differential data were visualized using R. Hallmark gene set and principal component analyses (PCA) were performed in R and visualized in Prism GraphPad (version 9).

## 3 RESULTS

### 3.1 Whole organ perfusion

Donor characteristics of livers undergoing whole organ hyperthermic machine perfusion (*n* = 8) and normothermic machine perfusion (*n* = 8) are reported in Table 1. During hyperthermic whole organ machine perfusion at 40°C, we observed no distinct changes in perfusion parameters. There was a non‐significant uptrend in hepatic artery (HA) and portal vein (PV) flows during the temperature increase from 37°C to 40°C, continuing over the following 3 h (Figure 1c,d, respectively). Intrahepatic resistance remained unchanged (Figure 1e). During the warming phase from 37°C to 40°C, lactate levels slightly increased in both arterial (0.89–1.15 mmol/L; *p* = 0.045) and venous (0.96–1.23 mmol/L; *p* = 0.034) samples and gradually returned to baseline levels over the subsequent 3 h (Figure 1f). Similarly, a significant increase in oxygen consumption was observed during the temperature increase from 37°C to 40°C (*p* = 0.025), suggesting a rise in respiratory rate and oxidative metabolism (Figure 1g). All machine perfusion parameters and blood gas results for arterial and venous measurements are provided in the supplementary materials (Figures S1–S3, respectively) and show non‐significant changes over time.

**TABLE 1:** Donor characteristics for livers included in whole organ hyperthermic perfusion.

	All (*n* = 16)	Normothermia (control) (*n* = 8)	Hyperthermia (*n* = 8)	*P*‐value
Donor characteristics				
Age (years)	65.5 (62.75–70.25)	65.5 (64.25–70.5)	66 (62.75–68.75)	0.518
Body mass index (kg/m^2^)	24.9 (23.9–25.9)	23.9 (22.5–24.9)	25.705 (24.6–27.1)	0.070
Gender				1.000
Male	10 (62.5%)	5 (62.5%)	5 (62.5%)	
Female	6 (37.5%)	3 (37.5%)	3 (37.5%)	
Cause of death				0.032
Trauma	4 (25%)	2 (25%)	2 (25%)	
CVA	5 (31.25%)	0 (0%)	5 (62.5%)	
Anoxia	5 (31.25%)	4 (50%)	1 (12.5%)	
Other	2 (12.5%)	2 (25%)	0 (0%)	
Donor type				
DCD	16 (100%)	8 (100%)	8 (100%)	1.000
DBD	0 (0%)	0 (0%)	0 (0%)	
Time from withdrawal of life support to circulatory arrest (min)	14 (10–20)	10 (7.5–11)	18.5 (14–24.5)	0.365
Time from circulatory arrest to cold perfusion (min)	17 (15–19)	18 (14.5–22)	17 (16.5–19)	0.374
Functional donor warm ischemia time^a^ (min)	22 (15–29)	25 (22–47.5)	17.5 (13–23.25)	0.164
Last sodium (mmol/L)	144 (140.5–149)	142.5 (140–143.75)	147 (144–149)	0.191
Last AST (U/L)	48.5 (35–69.5)	60 (42.5–76)	37 (25.25–62)	0.212
Last ALT (U/L)	53.5 (25.25–71.75)	66.5 (64–108.25)	24.5 (18.75–32.25)	0.062
Last GGT (U/L)	78 (38.75–108.75)	92.5 (82–134)	44 (28.25–78)	0.141
Hepatectomy time (min)	34 (28–39)	36 (31.75–39.5)	32 (27.5–37.5)	1.000
Static cold ischemia time (min)	249 (222.75–284.25)	220.5 (191.25–239.75)	284 (252–287.25)	0.018
DRI^b^	3.057 (2.716–3.246)	3.0555 (2.716–3.274)	3.0605 (2.721–3.219)	0.961

*Note*: Continuous data are presented as median (IQR), categorical data as a number (percentage).

Abbreviations: ALP, alkaline phosphatase; ALT, alanine aminotransferase; AST, aspartate aminotransferase; CVA, cerebral vascular accident; DBD, donation after brain death; DCD, donation after circulatory death; DRI, donor risk index; GGT, gamma glutamyl transferase.

^a^ Time from donor saturation <80% or mean arterial pressure <60 mm Hg to initiation of in‐situ cold flushing in the donor. Static cold ischemia time was defined as the time between initiation of cold flushing in the donor and start of DHOPE.

^b^ Validated scoring tool to assess the risk of liver graft failure.

### 3.2 Whole organ perfusate reveals an increase in lipid metabolism and heat shock proteins over time, but a minimal response to hyperthermia

To identify possible changes in protein abundance related to temperature changes and over time, we used an untargeted, DIA mass spectrometry approach. Across 64 perfusate samples from 16 whole organ perfusions, we identified 2519 unique proteins.

**FIGURE 2:**
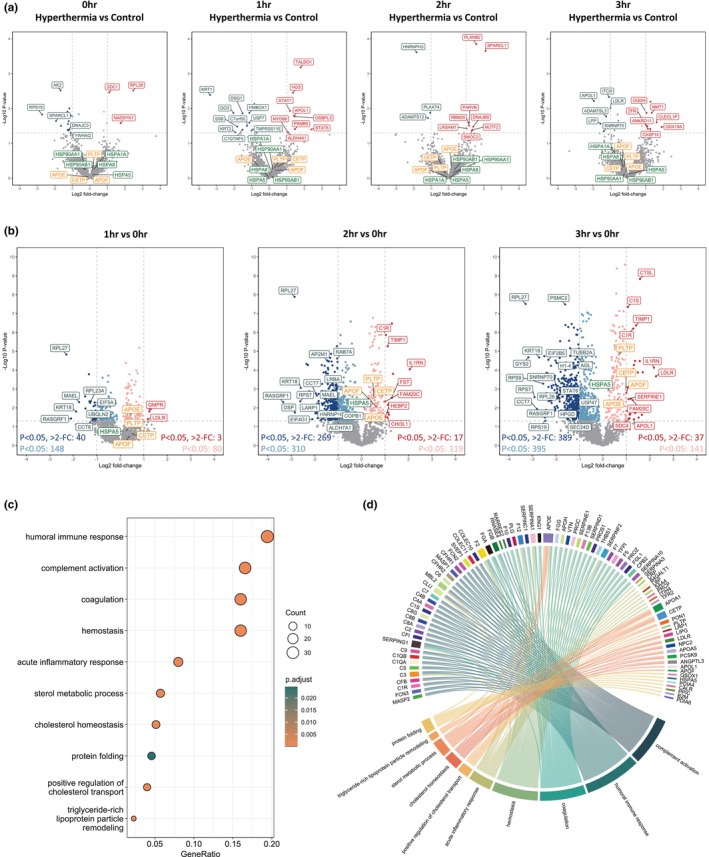
Proteomics analysis in perfusate from whole organ hyperthermic perfusion (*n* = 8) and normothermic perfusion (*n = 8*). (a) Volcano plots showing significance and fold change of protein intensity in perfusate of hyperthermic versus normothermic perfusion at 0, 1, 2 and 3 h. Statistics were performed using a two‐tailed Students *t*‐test with a permutation‐based FDR of 0.05 to test multiple comparisons. Significant proteins (*p* < 0.05, >2‐fold change) are highlighted red for upregulated (enriched in hyperthermia) and blue for downregulated (enriched in normothermia). Proteins that were significant but did not reach >2‐fold change are highlighted in light red/blue. Proteins involved in lipid metabolism and heat shock protein responses are highlighted in orange and green, respectively. (b) Volcano plots showing significance and fold change of protein intensity in perfusate over time at 1, 2 and 3 versus 0 h. Statistics were performed using a two‐tailed Students *t*‐test with a permutation‐based FDR of 0.05 to test multiple comparisons. Significant proteins (*p* < 0.05, >2‐fold change) are highlighted red for upregulated (enriched at 1, 2 or 3 h) and blue for downregulated (enriched at 0 h). Proteins that were significant but did not reach >2‐fold change are highlighted in light red/blue. Proteins involved in lipid metabolism and heat shock protein responses are highlighted in orange and green, respectively. (c) Dot plot depicting functional enrichment of gene ontology biological processes relating to significant (*p* < 0.05) proteins after 3h of perfusion. (d) Circos plot showing protein associations with significant gene ontology biological processes at 3 h of perfusion.

Differential analysis of the identified proteins showed that hyperthermia resulted in minimal change when compared to control samples perfused at 37°C at 0, 1, 2, and 3 h (Figure 2a). The number of significantly abundant proteins (*p* < 0.05) enriched in hyperthermia increased from 6 to 10 at 0 and 3 h, respectively. Of these proteins, 2‐Oxoglutarate Dehydrogenase (OGDH) is a key enzyme in the TCA cycle, used for modulating cellular energy metabolism (Jiahui et al., 2025). Furthermore, N‐myristoyltransferase 1 (NMT1) has been shown to influence lipid modifications (Hannoush & Sun, 2010). However, no significant gene ontology pathways were identified as being enriched in the hyperthermia condition at any time point. Highlighted lipoproteins and HSPs also showed no significant increase in the hyperthermia group compared to controls over 3 h of perfusion.

Due to minimal changes between groups, we performed differential analysis of the identified proteins in all samples over time. This showed that the perfusate exhibited a moderate but continually increasing number of significant (*p* < 0.05) protein IDs over time when compared to baseline. The number of significantly differentially abundant proteins increased from 188 to 784 for downregulated proteins and from 83 to 178 for upregulated proteins at 1 and 3 h, respectively. This upward trend suggests progressive responses over the course of the perfusion. However, most of these proteins showed <2‐fold change in abundance (Figure 2b). Given that far fewer proteins displayed both statistical significance and >2‐fold change, we performed functional enrichment analysis on all proteins that were differentially significant (*p* < 0.05 only). Throughout the 3‐h period of perfusion, we observed significant upregulation of processes related to immune response (*p* = 0.008 at 1 h, *p* = 2.09E‐18 at 3 h), hemostasis (*p* = 1.11E‐12 at 1 h, *p* = 6.851E‐21 at 3 h) and coagulation (*p* = 8.79E‐13 at 1 h, *p* = 4.41E‐21 at 3 h), indicating an active attempt of the liver to regulate inflammatory responses and maintain homeostasis.

At 3 h of perfusion, functional enrichment analysis revealed further significant upregulation of processes related to protein folding (*p* < 0.001) as well as lipid‐related pathways including sterol metabolism (*p* = 0.002), cholesterol transport (*p* < 0.001) and triglyceride‐rich protein remodeling (*p* < 0.001) (Figure 2c). We next used a circos analysis to better visualize the relationship between proteins and their associated pathways. This identified a clear subset of lipoproteins involved in the enriched cholesterol and other metabolic processes (Figure 2d).

To better understand the time‐dependent changes of proteins involved in cholesterol metabolic processes, we further examined the differential analysis shown in Figure 2b. Early and significant upregulation of apolipoprotein APOE (*p* = 0.028) was observed as early as 1 h. In addition, we observed a progressive increase in the significance of cholesteryl ester transfer protein (CETP, *p* = 0.220 to *p* < 0.001) and phospholipid transfer protein (PLTP, *p* = 0.182 to *p* < 0.001) over time. Although the change for these proteins was less than two‐fold, their consistent upregulation suggests an important role in lipid transport and remodeling during perfusion.

In addition to lipid metabolism, protein folding processes also showed significant upregulation after 1 h (*p* = 0.004) and continued to 3 h of perfusion (*p* = 0.002). Furthermore, HSPA5 (member of the HSP70 family) showed significant upregulation after 2 h of perfusion (*p* = 0.013) and remained significant at 3 h (*p* = 0.001), despite low fold change (Figure 2b).

Taken together, the proteomic response to hyperthermia in the perfusate is minimal over the 3 h of perfusion. Time‐dependent changes elicited a much more pronounced proteomic response, characterized by significant changes in lipoprotein abundance, cholesterol homeostasis, lipoprotein particle remodeling, and protein folding mechanisms.

### 3.3 Lipoprotein profiling in perfusate indicates increased lipid export over time

Given the significant upregulation of lipid metabolism pathways observed in the proteomic analysis of perfusate over time, we sought to further explore hepatic lipid output following whole organ hyperthermic perfusion, using direct analysis of triglycerides, cholesterol, and apolipoproteins in the perfusate.

**FIGURE 3:**
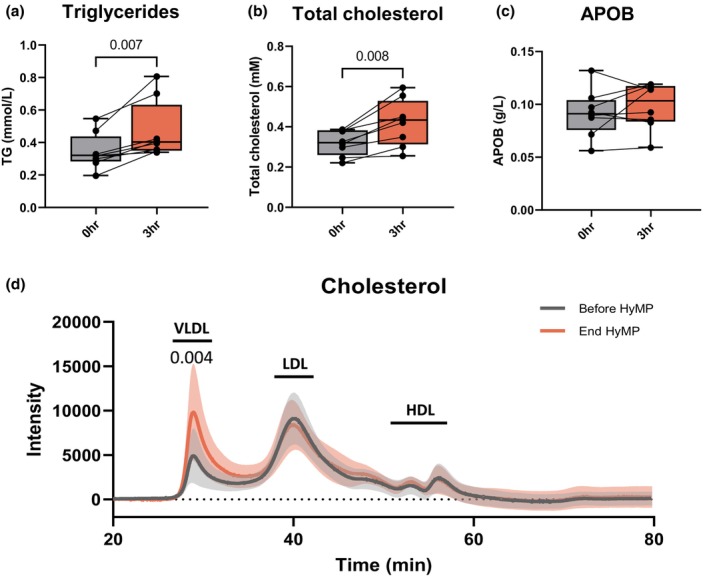
Lipoprotein profiling in whole organ perfusate (*n* = 8). (a) Triglycerides, (b) total cholesterol, and (c) APOB measured in whole organ machine perfusion perfusate before (0 h, 37°C) and at the end (3 h, 40°C) of hyperthermic perfusion. Box plots are presented as median ± min–max. (d) Lipid profiling of fast protein liquid chromatography (FPLC)‐separated lipoprotein fractions in perfusate. Significance was determined by defining the peaks (VLDL; 26.3–34.8 min) to assess area under the curve (AUC) for each whole organ experiment. AUCs were then analyzed using a paired *t*‐test. Lines and boundaries denote mean value with standard deviation.

Our analysis revealed a significant increase in triglycerides (*p* = 0.007) and cholesterol (*p* = 0.008) after hyperthermia (Figure 3a,b). Given that the liver typically lipidates APOB with triglycerides and cholesterol to form very low‐density lipoproteins (VLDL), which are subsequently secreted into the bloodstream, we next assessed direct APOB concentration and assessed the perfusate for the anticipated presence of lipoproteins using FPLC to examine whether this pathway was influenced by hyperthermia. While the perfused livers produced APOB, there was no significant change in APOB protein concentration after 3 h of hyperthermia when compared to baseline (Figure 3c). This finding is consistent with the proteomics data, where APOB did not show significant changes in abundance over time. Lipoprotein profiling (Figure 3d) showed that the perfused liver produced particles of varying sizes, including VLDL‐sized particles, as well as low‐density lipoprotein (LDL) and high‐density lipoprotein (HDL)‐sized particles. Notably, hyperthermia induced a significant increase in the concentration of VLDL‐sized particles, whereas there were no significant changes in the levels of LDL or HDL‐sized particles. These data combined suggest that hyperthermia induces an increase in hepatic lipoprotein abundance that is associated with an increase in VLDL‐sized particles.

### 3.4 Hyperthermic perfusion has minimal effect on the tissue proteome after 3 h

Building on the protein changes observed in perfusate, we sought to examine whether similar trends could be observed in liver tissue biopsies taken from the same perfusions. We performed the same proteomics analysis on 32 tissue samples collected at 0 (37°C), 1, 2, and 3 h (40°C) from 8 individual whole organ hyperthermic perfusions. Across these samples, we identified 5794 unique proteins.

**FIGURE 4:**
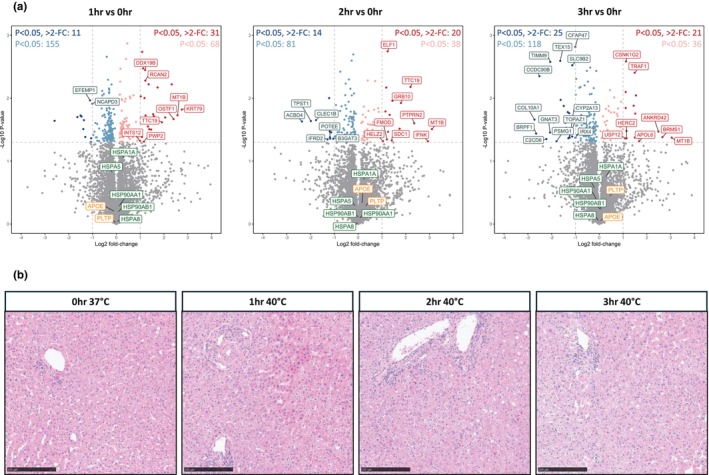
Proteomics in tissue from whole organ hyperthermic perfusion (*n* = 8). (a) Volcano plots showing significance and fold change of protein intensity in perfusate at 1, 2 and 3 versus 0 h. Statistics were performed using a two‐tailed Students *t*‐test with a permutation‐based FDR of 0.05 to test multiple comparisons. Significant proteins (*p* < 0.05, >2‐fold change) are highlighted red for upregulated (enriched at 1, 2 or 3 h) and blue for downregulated (enriched at 0 h). Proteins that were significant but did not reach >2‐fold change are highlighted in light red/blue. Proteins involved in lipid metabolism and heat shock protein responses are highlighted in orange and green, respectively. (b) H&E histological staining of whole organ tissue after 0 h (37°C), 1, 2 and 3 h (40°C). Scale bars 250 μm.

Differential analyses to identify significant (*p* < 0.05) changes in protein abundance revealed that hyperthermic perfusion had a minimal impact on the tissue proteome. Despite the changing profiles over the 3‐h perfusion, the total number of upregulated proteins, both significant (*p* < 0.05) and significant with more than a two‐fold change, remained relatively constant, finding only 99, 58, and 57 significantly upregulated proteins at 1, 2, and 3 h, respectively (Figure 4a). Further functional analysis did not reveal any significantly enriched biological processes linked to the differentially abundant proteins. This indicates that while the tissue proteome was not entirely static, the biological impact of these changes was minimal in terms of activating distinct pathways during this timeframe.

Given the importance of lipid metabolism, cholesterol transport, and HSP responses observed in the perfusate analysis, we specifically examined the abundance of proteins associated with these processes in the tissue samples. However, none of these proteins displayed significant changes throughout the 3‐h perfusion (Figure 4a). Furthermore, we observed no structural changes to liver tissue over 3 h of hyperthermic perfusion (Figure 4b), reinforcing that liver tissue remains relatively stable under ‘short term’, 3‐h hyperthermic conditions.

These findings suggest that while perfusion does induce notable changes in lipid metabolism and protein folding mechanisms in the perfusate over time, the tissue itself appears to maintain a steady‐state proteome with minimal perturbation within the 3‐h time window.

### 3.5 Precision‐cut liver slices show tissue proteome changes that occur after long‐term exposure to hyperthermic conditions

To investigate the longer‐term effects of hyperthermia on liver tissue at the molecular level beyond 3 h, we utilized PCLS from human donor livers. This allowed us to observe how sustained exposure to mild hyperthermic conditions influences the tissue proteome over an extended period of up to 48 h.

Initially, PCLS experiments were conducted on 24 human donor livers, and ATP content was measured at each timepoint (0, 3, 24, and 48 h) at both 37°C and 40°C to assess slice viability. Only PCLS that maintained viable ATP levels (ATP/protein >2 pmol/μg) were included in the study. After excluding 6 livers based on ATP viability criteria, PCLS from 18 livers were subjected to 3, 24, and 48 h of hyperthermic exposure (Figure 1a).

**FIGURE 5:**
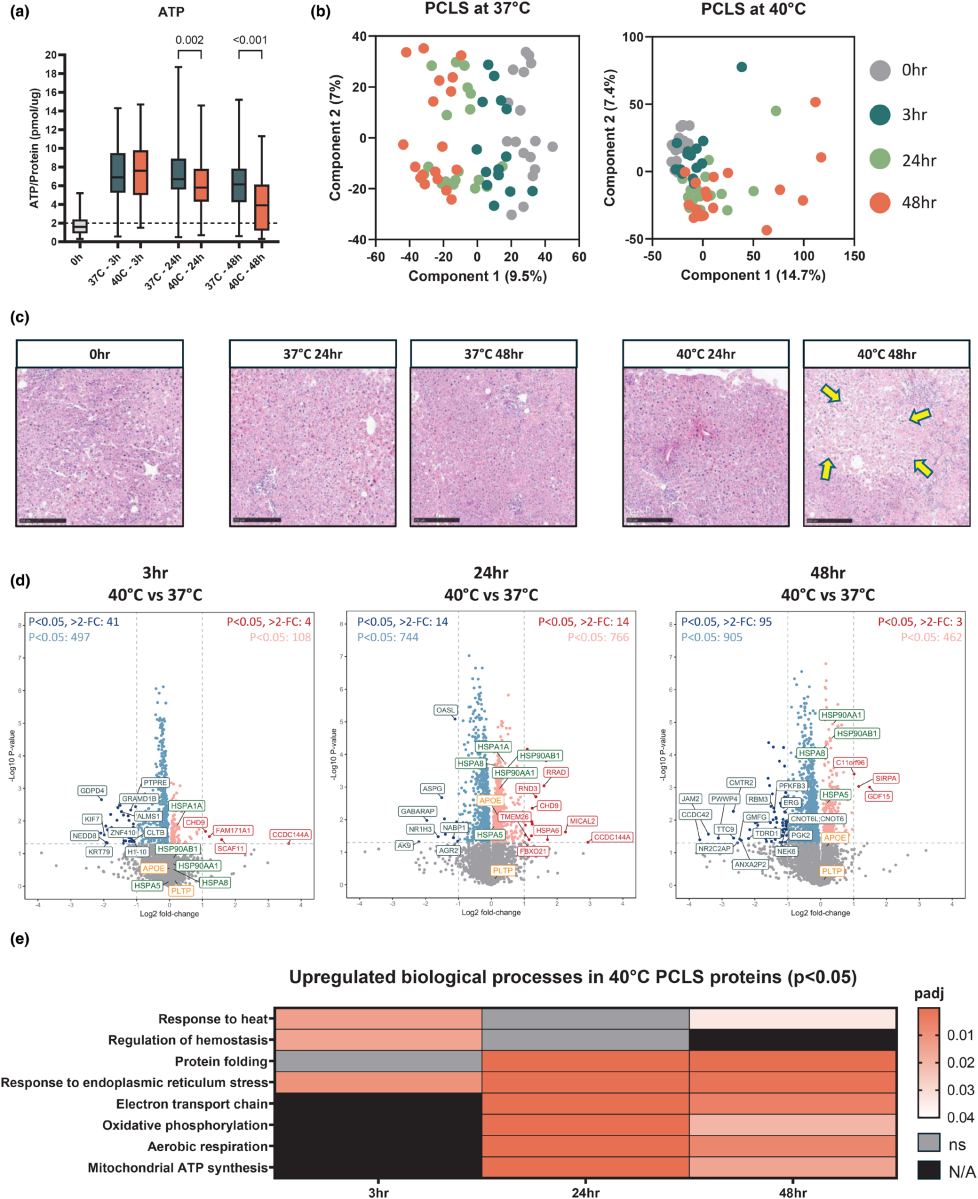
Proteomics analysis in tissue from precision‐cut liver slices (PCLS, *n* = 18). (a) Viability of PCLS were determined by ATP/protein (pmol/μg) content. Data are shown as median ± min–max; 3–6 slices were used in each experiment (*n* = 18). (b) Principal component analysis showing timepoint variation in PCLS and 37°C and 40°C. (c) H&E histological staining of PCLS after 0, 3, 24 and 48 h with incubation at 37°C and 40°C. Scale bars 250 μm. Arrows highlight patches of necrosis. (d) Volcano plots showing significance and fold change of protein intensity in perfusate at 3, 24 and 48 versus 0 h. Statistics were performed using a two‐tailed Students *t*‐test with a permutation‐based FDR of 0.05 to test multiple comparisons. Significant proteins (*p* < 0.05, >2‐fold change) are highlighted red for upregulated (enriched at 3, 24 or 48 h) and blue for downregulated (enriched at 0 h). Proteins that were significant but did not reach >2‐fold change are highlighted in light red/blue. Proteins involved in lipid metabolism and heat shock protein responses are highlighted in orange and green, respectively. (e) Heatmap of upregulated gene ontology biological processes at 3, 24 and 48 h in PCLS at 40°C. ns, not significant; N/A, pathway not identified.

The viability assessment using ATP measurements showed a gradual decline over time, with PCLS incubated at 40°C exhibiting significantly lower ATP values than those incubated at 37°C after 24 h (*p* = 0.002) and 48 h (*p* < 0.001; Figure 5a). PCA of PCLS protein profiles revealed timepoint‐associated clustering, with increased variability in samples incubated at 40°C, particularly after 48 h, reflecting a decline in PCLS viability (Figure 5b). Morphological assessment following H&E staining confirmed reduced viability of PCLS after 48 h through the presence of necrotic areas (Figure 5c).

To further explore the proteomic changes in PCLS incubated at 37°C and 40°C, we analyzed tissue proteomics over time. Differential analysis revealed a substantial number of significant (*p* < 0.05) changes in protein abundance between PCLS at different temperatures. The smallest proportion of changes occurred at 3 h, where 538 proteins were downregulated and 112 were upregulated (Figure 5d). The most pronounced changes occurred at 24 h, where the number of downregulated (758) and upregulated (780) proteins was nearly equal, indicating a significant shift in protein dynamics at this timepoint. After 48 h of hyperthermia, we observed a notable shift toward downregulation, with 1000 proteins downregulated and only 465 upregulated (Figure 5d). This pattern aligns with the observed decline in PCLS viability, indicating that sufficient time is required for measurable protein abundance changes to occur, but prolonged hyperthermia for 48 h leads to a sharp decline in protein upregulation. This is consistent with the significant loss of viability seen in ATP measurements and histology at this timepoint, and suggests that prolonged exposure to mild hyperthermia is detrimental to long‐term PCLS survival in this model.

As in the whole organ perfusion experiments, we focused on proteins associated with lipid metabolism and HSP responses. Similar to the findings in whole organ tissue proteomes, APOE and PLTP showed minimal change after 3 h (Figure 5d). However, APOE abundance became significantly different (*p* = 0.007) after 24 h of hyperthermia but declined again by 48 h. In contrast, while HSPs did not show significant changes in whole organ tissue proteomes, a variety of HSPs, including members of the HSP70 and HSP90 families, became significantly abundant in PCLS at 40°C after 24 h, with further increases observed after 48 h (Figure 5d). This suggests that the tissue's prolonged exposure to hyperthermic stress triggers a delayed but significant HSP response.

Functional enrichment analysis also reflected these trends. Processes related to the response to heat and regulation of hemostasis showed decreasing significance over time. In contrast, metabolic‐related pathways, including respiratory processes, oxidative phosphorylation, and mitochondrial ATP synthesis, shifted from being undetected at 3 h to highly significant at 24 h (Figure 5e). However, at 48 h, the significance of all processes declined, consistent with the reduced number of upregulated proteins and the overall reduction in PCLS viability at this time point. This was also observed in the analysis of hallmark gene sets. Despite none of the processes being statistically significant, a substantial up‐regulatory shift in process upregulation can be observed from 3 to 24 h. This shift begins to revert after 48 h, reflecting the functional enrichment analyses (Figure S4).

These findings demonstrate that while tissue‐level protein changes are minimal in the short term (3 h), longer term (24 h) exposure to hyperthermia induces significant alterations in the proteome. The delayed but substantial shifts in protein abundance, particularly in HSP responses and metabolic processes, suggest that hyperthermic stress exerts cumulative effects on liver tissue, becoming more pronounced over time.

### 3.6 Prolonged exposure to hyperthermic conditions promotes heat shock protein responses

To further investigate cellular responses to hyperthermic conditions, we conducted a detailed analysis of HSP abundance across the whole organ tissues and perfusates, and PCLS. HSPs play a critical role in maintaining cellular homeostasis under stress conditions, and their activation is a hallmark of the cellular response to heat stress.

**FIGURE 6:**
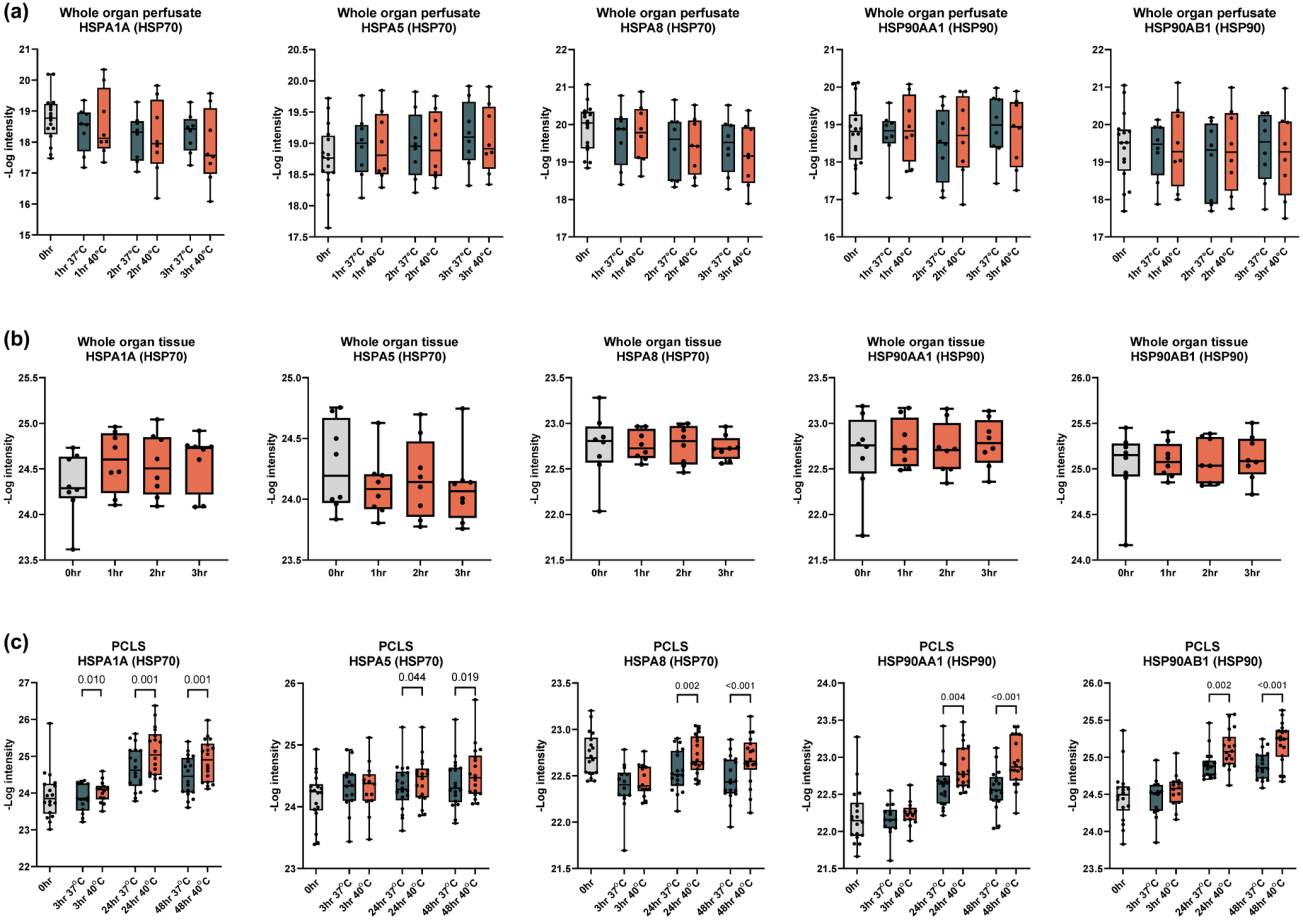
Heat shock protein (HSP) intensity in whole organ perfusion and PCLS. Protein intensity from LC–MS/MS analysis of HSP1A1, HSPA5, HSPA8, HSP90AA1, and HSP90B1 in (a) whole organ perfusate at 0, 1, 2, and 3 h at 37°C and 40°C, (b) whole organ tissue at 0, 1, 2, and 3 h, and (c) PCLS tissue at 0, 3, 24, and 48 h incubated at 37°C and 40°C. *p*‐values were calculated using one‐way ANOVA with Holm‐Šídák's multiple comparisons test. Data are shown as median ± min–max.

Our proteomics analysis identified more than 100 HSPs across 11 different HSP families in the tissue and perfusate of whole liver setups, as well as in PCLS. In the short‐term hyperthermic exposure (3 h), minimal changes were observed in the levels of HSPs in both the perfusate and tissue samples (Figure 6a,b), confirming the differential analysis (Figures 2a and 4a). Among the few exceptions, HSPA5 emerged as significantly upregulated in the perfusate after 2 h of perfusion (*p* = 0.013; Figure 2b); this was not statistically significant between hyperthermic and control groups (Figure 6a).

However, the most significant changes in HSP levels were observed in the longer‐term PCLS experiments. Notably, after 24 and 48 h of hyperthermic incubation at 40°C, HSP70 and HSP90 families showed marked upregulation compared to those incubated at 37°C. Specifically, HSPA1A, HSPA5, and HSPA8 (HSP70 family), as well as HSP90AA1 and HSP90AB1 (HSP90 family), exhibited significant increases in abundance (Figure 6c). These findings suggest a time‐dependent effect for measurable HSP response.

## 4 DISCUSSION

The shortage of available grafts for liver transplantation has led to an increased reliance on organs from ECD donors, including steatotic livers, which are more vulnerable to IRI. Advances in machine perfusion have significantly improved the preservation and utilization of ECD livers, providing a controlled environment for targeted interventions without systemic influences (Brüggenwirth et al., 2024; Nasralla et al., 2018; van Leeuwen et al., 2022; van Rijn et al., 2021). In this study, we investigated the effects of mild hyperthermic conditions (40°C) on human donor livers as a potential therapeutic intervention for ECD grafts. We observed moderate proteomic changes in both machine perfusion perfusate and liver tissue, alongside a significant increase in triglycerides and VLDL‐sized particles during whole organ perfusion. Additionally, prolonged exposure to hyperthermic conditions in PCLS revealed a significant increase in HSP70 and HSP90 family members after 24 and 48 h as a direct result of hyperthermic conditions over longer periods.

As proteomic analysis in this study was conducted on perfusate collected during machine perfusion, comparability between groups was based on viability status at the time of assessment. Viability was determined using both hepatocellular (perfusate) and cholangiocellular (bile) biochemical criteria (Thorne et al., 2023; van Leeuwen et al., 2025). All included livers met hepatocellular viability thresholds but failed on biliary grounds, making them biologically suitable for comparison. Moreover, no major differences were observed in donor characteristics between the groups, aside from variability in factors such as cause of death and cold ischemic time, the duration of which remained clinically acceptable for both groups (Table 1). These considerations support that any observed proteomic differences are more likely attributable to biological variation rather than baseline bias.

Fatty livers are known to lose their fat after several days in the recipient (Doyle et al., 2010). Previous studies in steatotic porcine livers have shown up to 50% reduction in fat content after 48 h of NMP (Jamieson et al., 2011). However, recreating this in a human setting has been challenging (Banan et al., 2016; Da Silva et al., 2023). As machine perfusion lacks systemic influences, enhancing the rate of metabolic activity and intercellular fat loss may be key in machine perfusion of human livers. Moreover, machine perfusion has the added benefit of managing fat loss in a controlled setting, potentially improving microcirculation and reducing peri‐ and post‐transplant complications associated with steatotic livers (Patrono et al., 2023, 2024). Current therapeutic approaches during machine perfusion, such as the use of ‘defatting cocktails’, have shown rapid decreases in hepatocellular fat content, but the long‐term effects of these interventions remain unknown (Abbas et al., 2024; Boteon et al., 2019; Li et al., 2023).

In this study, mild hyperthermic perfusion induced marked changes in metabolism. We observed a significant transient increase in oxygen consumption and lactate levels, correlating with the temperature rise and indicating heightened metabolic demand. Furthermore, we observed a significant increase in triglycerides and cholesterol in perfusate after 3 h, accompanied by a rise in VLDL‐sized particles, as confirmed by FPLC. Despite unchanged levels of APOB, the increase in VLDL‐sized particles suggests improved lipidation of APOB proteins, facilitating more efficient lipid export from the cell under short‐term hyperthermic conditions leading to the measured increase in perfusate cholesterol levels. These processes include apolipoproteins such as APOE and APOF, which are involved in lipid transport and clearance. APOE facilitates the binding, uptake, and clearance of lipoproteins by the liver, while APOF plays a role in regulating lipid metabolism through its interaction with other apolipoproteins (Huang & Mahley, 2014). In addition, CETP and PLTP are important in lipid transfer processes, with CETP mediating the exchange of cholesteryl esters and triglycerides between lipoproteins and PLTP promoting phospholipid transfer between lipoproteins (Jiang et al., 2012). These proteins work together to regulate lipid metabolism and lipoprotein remodeling, further supporting the observed increase in VLDL particles under hyperthermic conditions. The observed upregulation of CETP and APOF could be due to changes at the mRNA level or be caused by differences in hepatic secretion versus re‐uptake of lipoproteins, as both proteins are associated with secreted lipoproteins. In the absence of peripheral VLDL catabolism—normally mediated primarily by lipoprotein lipase in peripheral tissues (heart, skeletal muscles and adipose tissue), the direct cause of these results is currently unknown and requires further investigation. VLDL metabolism is a highly dynamic process influenced by a variety of processes including hormonal regulation primarily through glucagon and insulin. In physiological conditions, VLDL triglycerides are predominantly derived from peripheral fatty acids, as well as lysosomal triglyceride lipolysis, de novo lipogenesis, and triglyceride mobilization from cytosolic lipid droplets. Given that our ex‐situ perfusion model lacks peripheral fatty acid influx, the increased triglycerides detected in perfusate must have originated from an alternative pathway. However, the precise mechanisms underlying this lipid mobilization, devoid of systemic influences, remain unclear. While the presented H&E stains do not indicate severe steatosis in these livers, hyperthermic machine perfusion could still present a valuable therapeutic strategy, particularly in livers with high fat content. All livers, even macroscopically lean organs or those from individuals with normal to low BMI, contain intracellular fat deposits. The livers included in this study were limited to those initially declined for transplantation and underwent NMP viability assessment or were offered directly for research purposes and therefore were not specifically steatotic. Nonetheless, the observed increase in VLDL export may facilitate intracellular fat loss over prolonged perfusion durations, which could prove particularly beneficial for steatotic organs.

Perfusion of liver grafts at mild hyperthermic temperatures represents a moderate, natural alternative to pharmacological interventions, with the added benefit of activating protective HSP responses (Aufricht, 2005; Kalmar & Greensmith, 2009; O'Neill et al., 2014). HSPs are produced in response to a variety of cellular stresses and act as chaperones that aid in the folding of new proteins and refolding of denatured or misfolded proteins (Vabulas et al., 2010; Wang et al., 2014). The increase in temperature during hyperthermic perfusion induces endoplasmic reticulum (ER) stress, leading to the activation of the unfolded protein response. This stress response is a key regulator of cellular proteostasis, triggering pathways to restore protein homeostasis by enhancing the expression of molecular chaperones such as HSPs. These proteins play a crucial role in mitigating ER stress by stabilizing unfolded or misfolded proteins, preventing aggregation, and facilitating their proper refolding or degradation (Xu et al., 2011). We found that, although minimal significant differences in HSP protein abundances were present during 3 h of whole organ perfusion, extended exposure of PCLS to hyperthermic conditions resulted in significant HSP‐family protein abundances after 24 h. Transient, 10‐min bursts of hyperthermia have previously been shown to induce HSP70 mRNA transcripts in perfused rat livers, which contributed to the mitigation of IRI injury upon reperfusion (von Horn & Minor, 2020). Extended exposure of PCLS to hyperthermic conditions resulted in significant increases in HSP family proteins, particularly HSPA1A, HSPA5, and HSPA8 (HSP70 family), and HSP90AA1 and HSP90AB1 (HSP90 family) after 24 and 48 h. This suggests that while shorter periods of hyperthermia may initiate heat shock responses at the transcript level, longer exposure is required to observe significant protein‐level changes. The decline in cellular viability and metabolic processes observed after 48 h indicates that a 24‐h period of hyperthermic perfusion may be optimal for inducing beneficial cellular responses without overstressing the organ. Our findings highlight the importance of duration in hyperthermic treatments. Prolonged exposure to hyperthermic conditions, particularly over a 24‐h period, appears necessary to trigger significant HSP production and metabolic changes. Therefore, hyperthermia could be applied during long‐term perfusion protocols for multiple days, allowing for sustained interventions that could rehabilitate ECD organs and prepare them for transplantation (Da Silva et al., 2023; Lascaris et al., 2022). Building on our PCLS findings, hyperthermic exposure during whole organ machine perfusion may be more effective when extended to 24 h, followed by a return to normothermia, rather than being limited to a short 3‐h window. However, the duration of hyperthermic exposure must be carefully optimized, as our data indicate that prolonged exposure beyond 24 h may become detrimental to liver tissue. An alternative strategy to enhance the benefits of hyperthermia could involve increasing the temperature, although this approach poses risks, as temperatures above 41.5°C are known to significantly impact cellular viability (Hildebrandt et al., 2002). Moreover, maintaining a stable perfusion temperature close to this threshold, particularly for long periods, presents technical challenges and may inadvertently damage the organ. In the case of extended machine perfusion over several days, the addition of a fat filter in long‐term machine perfusion setups may also be essential to prevent lipoprotein accumulation and protect the oxygenators from clogging, while facilitating efficient lipid export from the liver.

Limitations of this study include the lack of transplantation in the whole organ perfusion model, preventing assessment of the effect of mild hyperthermia on IRI in the recipient. Additionally, termination of the whole organ perfusion model at 3 h limits our understanding of long‐term hyperthermic effects in the whole organ. Future studies should, based on our results, investigate the effect of mild hyperthermic pulses lasting up to 24 h within extended perfusion protocols over several days, rather than a single sustained increase. A pulsed hyperthermic treatment over several days may be beneficial for inducing measurable HSP production and increasing metabolic rate without overstressing the organ. Such an approach could expedite fat loss more efficiently than passive methods and leverage the benefits of HSP induction. Due to the limited availability of suitable whole organs for experimentation, only perfusate samples were available for direct proteome comparison between experimental and control groups. Direct tissue comparison was not possible due to concerns that additional biopsies may result in excessive injury and potential bleeding during transplantation. Similarly, due to limited availability of whole organs for experimentation, we used PCLS as an alternative model to explore metabolic changes under hyperthermic conditions for longer periods of time. Despite these two models being inherently different, this approach helped mitigate the constraints in studying whole organs while still providing additional insight into the metabolic effects of hyperthermia.

In conclusion, exposure of isolated human livers to mild hyperthermic conditions may represent a viable therapeutic strategy for steatotic organs. By balancing the benefits of increased metabolic activity and protective HSP mechanisms without overstressing the liver, hyperthermia could improve the utilization of high‐risk liver grafts in transplantation.

## AUTHOR CONTRIBUTIONS

AMT conceived and designed research, performed experiments, analyzed data, interpreted results of experiments, prepared figures, drafted the manuscript and edited and revised the manuscript. YG, VAL, and MS performed experiments and edited and revised the manuscript. JAK, RJP, FK, PO, and JCW participated in research design and edited and reviewed the manuscript. VEM conceived and designed research, interpreted results of experiments, drafted manuscript, and edited and revised themanuscript.

## FUNDING INFORMATION

VM reports a VENI research grant by the Dutch Research Council (NWO; grant #09150161810030), a Research grant from the Dutch Ministry of Economic Affairs (Health~Holland Public Private Partnership grant #PPP31 2019‐024), and a Research grant from the Dutch Society for Gastroenterology (NVGE #01‐2021), all outside the submitted work. FK is supported by an unrestricted grant from the Noaber Foundation, Lunteren, The Netherlands.

## CONFLICT OF INTEREST STATEMENT

The authors declare no competing interests.

## ETHICS STATEMENT

This research complies with all ethical regulations. The use of human material was in accordance with Dutch legislation and the Code of Conduct for dealing responsibly with human tissue in the context of health research issued by the Committee on Regulation of Health Research, the COREON Foundation. Written informed consent for the use of donor livers for research purposes was obtained from the donor's relatives. The declarations of Istanbul and Helsinki were adhered to.

## Supporting information


Data S1.

## Data Availability

Raw mass spectrometry data used for proteomic analysis are available through ProteomeXchange via PRIDE, with the identifier PXD056736 (https://www.ebi.ac.uk/pride/archive/projects/PXD056736) (Perez‐Riverol et al., 2022). All other data are available in the main text or supplementary materials (10.6084/m9.figshare.28495418).
