# Supplementary material for: Therapeutic hyperthermia promotes lipid export and HSP70/90 during machine perfusion of human livers
Source: Physiol Rep. 2025 May 9;13(9):e70348. doi: 10.14814/phy2.70348 (PMC12064339; doi:10.14814/phy2.70348)
Supplement: Supplementary file 1 — Data S1. [file PHY2-13-e70348-s001.pdf]

Therapeutic hyperthermia promotes lipid export and HSP70/90 during machine perfusion of human livers

Supplementary materials

Supplementary figure 1. Parameters for whole organ hyperthermia machine perfusion.

Supplementary figure 2. Arterial biochemical blood gas measurements in whole organ hyperthermic machine perfusion.

Supplementary figure 3. Venous biochemical blood gas measurements in whole organ hyperthermic machine perfusion.

Supplementary figure 4. Hallmark gene set analysis.

Supplementary Figure 1

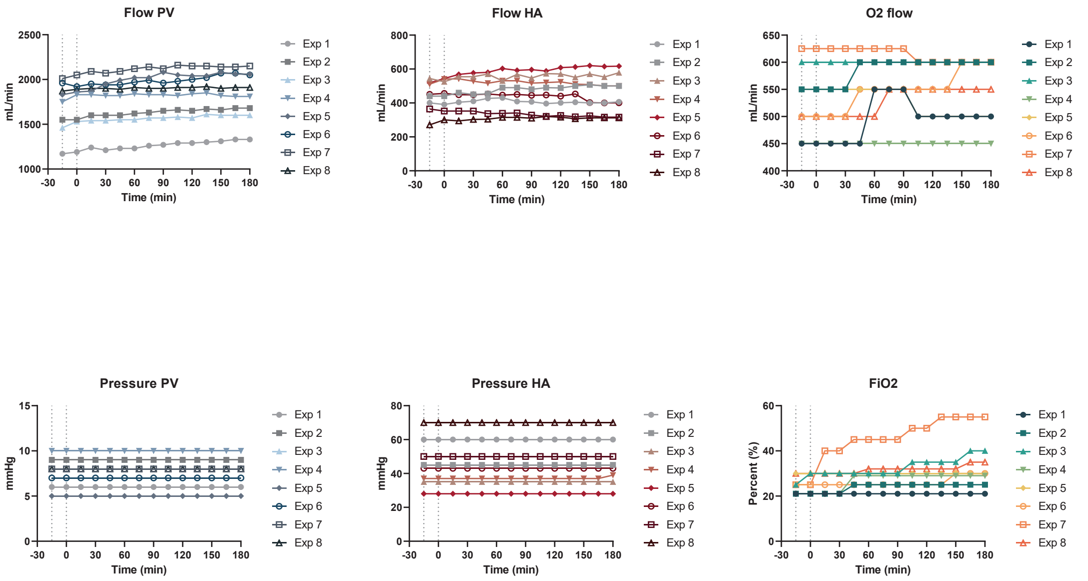

Supplementary Figure 1. Parameters for whole organ hyperthermia machine perfusion. Flow, pressure and oxygen parameters for machine perfusion of whole organs over 3 hours. Temperature increase occurred from -15 mins (37ºC) to 0 min (40ºC). HA; hepatic artery (red), PV; portal vein (blue).

Supplementary Figure 2

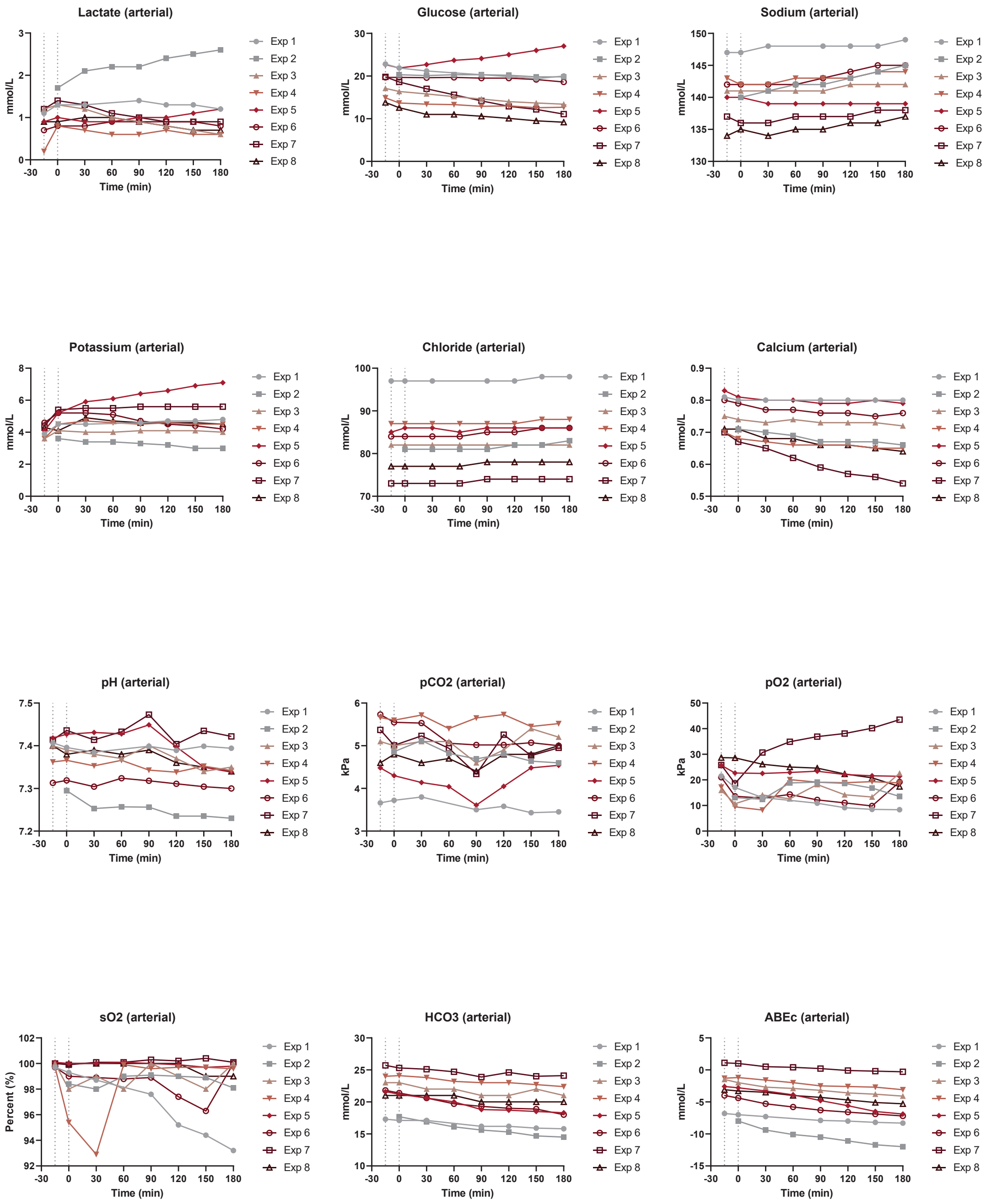

Supplementary Figure 2. Arterial biochemical blood gas measurements in whole organ hyperthermic machine perfusion. Temperature increase occurred from -15 mins (37°C) to 0 min (40°C).

Supplementary Figure 3

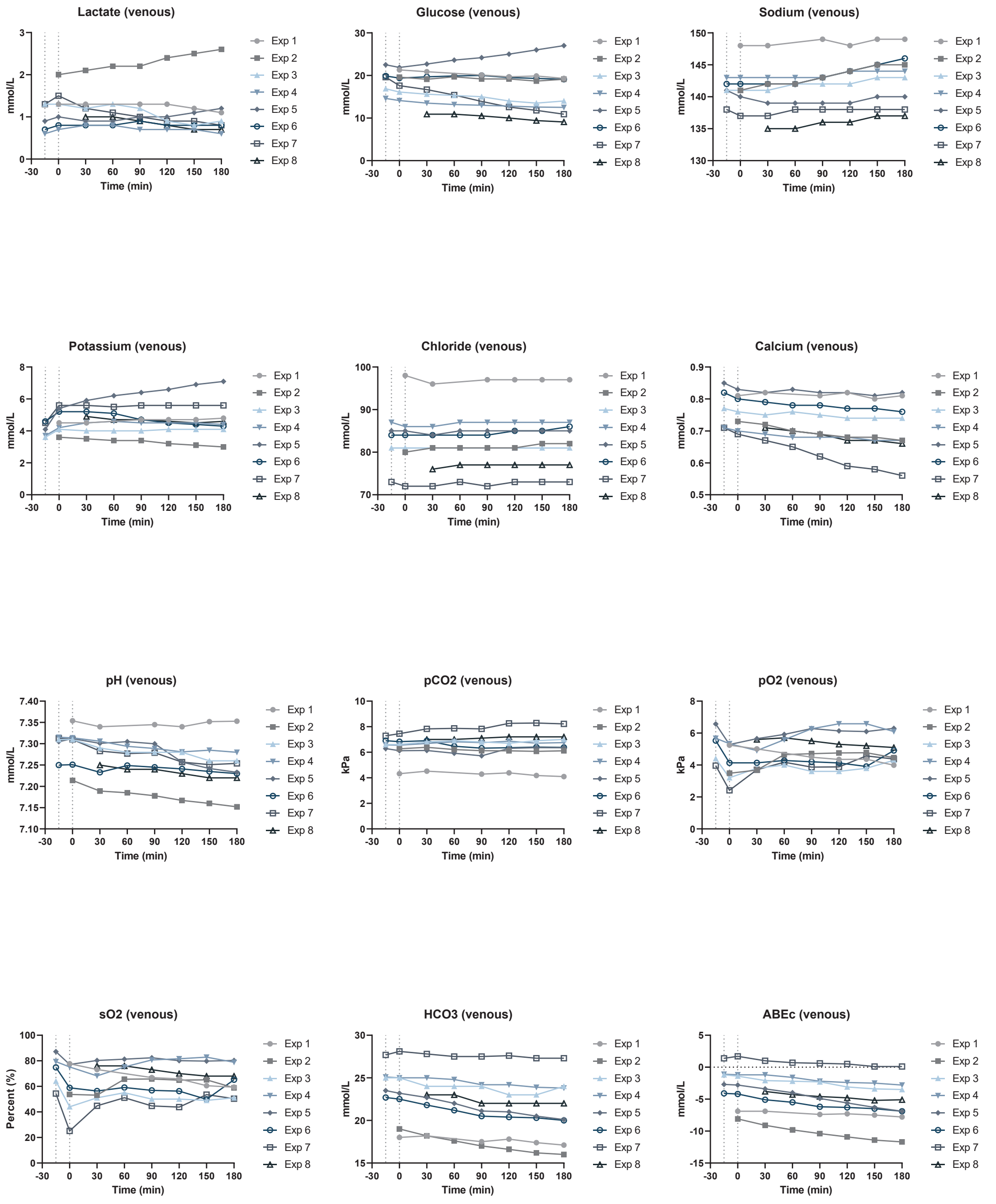

Supplementary Figure 3. Venous biochemical blood gas measurements in whole organ hyperthermic machine perfusion. Temperature increase occurred from -15 mins (37°C) to 0 min (40°C).

Supplementary Figure 4

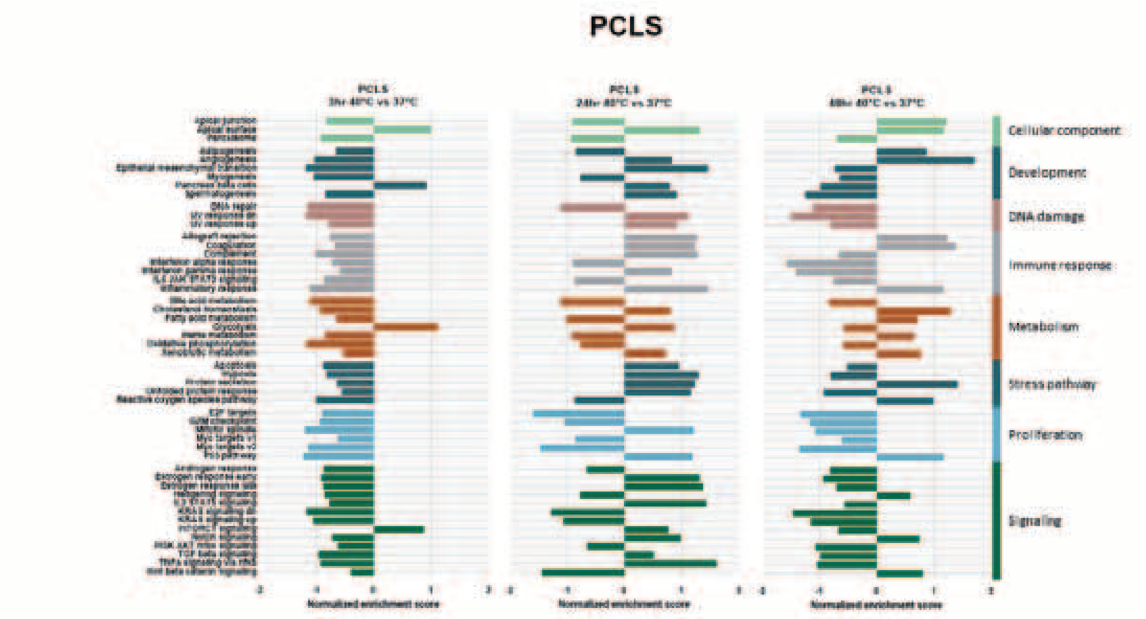

Supplementary Figure 4. Hallmark gene set analysis. Hallmark gene set analysis from LC-MS/MS proteomics in precision-cut liver slices (PCLS) of 40°C vs 37°C at 3hr, 24hr and 48hr. Gene sets are grouped and colored according to process. None of the process enrichments in PCLS reached statistical significance ($p<0.05$).
